# Heavier Load Alters Upper Limb Muscle Synergy with Correlated fNIRS Responses in BA4 and BA6

**DOI:** 10.34133/cbsystems.0033

**Published:** 2023-05-31

**Authors:** Zhi Chen, Jin Yan, Xiaohui Song, Yongjun Qiao, Yong Joo Loh, Qing Xie, Chuanxin M. Niu

**Affiliations:** ^1^ Department of Rehabilitation Medicine, Ruijin Hospital, Shanghai Jiao Tong University School of Medicine, Shanghai 200025, China.; ^2^ School of Medicine, Shanghai Jiao Tong University, Shanghai 200025, China.; ^3^ Department of Rehabilitation Medicine, Tan-Tock-Seng Hospital, Singapore.

## Abstract

In neurorehabilitation, motor performances may improve if patients could accomplish the training by overcoming mechanical loads. When the load inertia is increased, it has been found to trigger linear responses in motor-related cortices. The cortical responses, however, are unclear whether they also correlate to changes in muscular patterns. Therefore, it remains difficult to justify the magnitude of load during rehabilitation because of the gap between cortical and muscular activation. Here, we test the hypothesis that increases in load inertia may alter the muscle synergies, and the change in synergy may correlate with cortical activation. Twelve healthy subjects participated in the study. Each subject lifted dumbbells (either 0, 3, or 15 pounds) from the resting position to the armpit repetitively at 1 Hz. Surface electromyographic signals were collected from 8 muscles around the shoulder and the elbow, and hemodynamic signals were collected using functional near-infrared spectroscopy from motor-related regions Brodmann Area 4 (BA4) and BA6. Results showed that, given higher inertia, the synergy vectors differed farther from the baseline. Moreover, synergy similarity on the vector decreased linearly with cortical responses in BA4 and BA6, which associated with increases in inertia. Despite studies in literature that movements with similar kinematics tend not to differ in synergy vectors, we show a different possibility that the synergy vectors may deviate from a baseline. At least 2 consequences of adding inertia have been identified: to decrease synergy similarity and to increase motor cortical activity. The dual effects potentially provide a new benchmark for therapeutic goal setting.

## Introduction

Deficits in motor performances may improve upon movement training for patients with neurological diseases, which include stroke [1], Parkinson’s disease [2], and cerebral palsy [3]. If mechanical loads (such as weights and elastic bands) are applied during training, then additional benefits may show in gait [4], muscle strength [5], and functionalities in the upper limbs [6]. The effect of adding mechanical load may be partly explained by non-neurological improvements, such as increases in muscle mass [7], muscle endurance [8], cardiometabolic fitness [9], etc. However, it is likely that the role of the mechanical load during training is mainly reflected in the neurological responses to loading [10].

How the peripheral nervous system reacts to mechanical loads has been documented with debates. On the one hand, electromyography (EMG) of upper limb muscles is affected by increases in load, manifested as prolonged activation of agonists and delayed bursts of antagonists [11] or an overall amplification of EMG [12]. On the other hand, muscle vectors remain robust under different dynamic conditions. For instance, the activation patterns of hindlimb muscles (e.g., rectus internus major and adductor magnus) of bullfrogs remain stable during inertial load perturbations [13]; in patients with stroke, the activation pattern of shoulder muscles (e.g., anterior deltoid and biceps) also persevered in various upper limb movements [14]. In a word, the literature seems to agree that adding load can cause traceable changes in muscle EMGs, but the modular control embedded in muscle patterns may not be altered.

The response of the central nervous system to mechanical loads varies according to the extent of movement, i.e., the range of motion and applied load of the movement [15]. In small-extent movements (e.g., squeeze or grip), functional magnetic resonance imaging studies have claimed that the activation in motor cortices correlates positively with force outputs [16,17]. Large-extent movements bear high relevance in clinical therapy, but mechanical loading in large-extent movements is less investigated perhaps because of inevitable trunk movements during imaging [18]. It has been argued that large-extent movements undergo a different motor control strategy compared with small-extent movements [19]. In general, evidence is present but not abundant on how the nervous system organizes during mechanical loading, especially how the central reactions are orchestrated with peripheral activities.

Functional near-infrared spectroscopy (fNIRS) is an emerging technology for brain imaging featured in high ecological validity [20], which makes fNIRS suitable for the detection of cortical responses with head and trunk movements [21]. A previous study has identified that, during large-extent upper limb movements, the cortical activations of motor-related regions correlate with increases in load inertia [22]. Additionally, the magnitude of load has been found to affect movement velocity, torque, and EMG response latency [23]. To further explore the relationship among load inertia, muscle patterns, and cortical activation, we collected cortical hemodynamic signals and surface EMGs during the same movements. In this study, we collected cortical hemodynamic signals and surface EMGs during the same movements. Our central hypothesis is that increased load inertia alters muscle synergies, and the similarity of these synergies may correlate with fNIRS cortical response. If the hypothesis is supported, then it would shed light on the neurological mechanism of resistance training, which potentially provides a new benchmark for therapeutic goal setting.

## Materials and Methods

### Participants

Twelve healthy subjects between 24 and 33 years old (26.6 ± 2.9; 9 males and 3 females) participated in this study. None of the participants had any history of neurological or musculoskeletal disorders. The study had been approved by the Ethics Committee. Each participant gave written consent before the experiment.

### Movement task

**Fig. 1.**
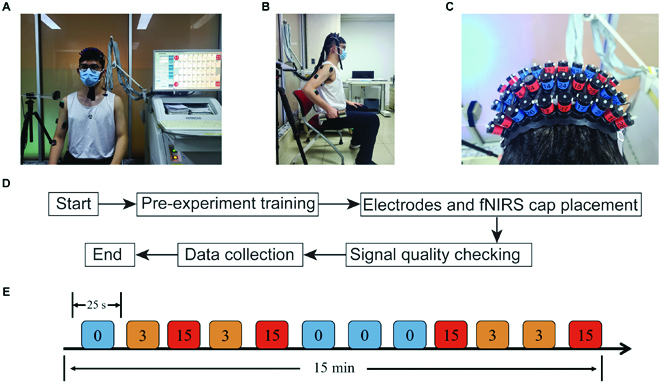
Experimental design. (A) The actual scene of the experiment. Subjects sat on the armless chair to allow the free movement of the upper limb. EMG sensors and the fNIRS probe were placed before the experiment. (B) The lifting movement, subjects were asked to lift the dumbbells to the armpit. (C) The 3 × 10 high-density fNIRS probe. Emitters were red and detectors were blue. The channels were separated 1.5 cm apart, fully covering motor-related regions (Brodmann Area 4 (BA4) and BA6) on both sides. (D) The flow chart of the experiment within one visit. (E) The timeline of the lifting experiment. 0 represents 0-pound condition (no extra load), etc.

As shown in Fig. 1A, each subject raised the right hand from the hanging position to the armpit while sat on an armless chair; both the forearm and upper arm were required to move in the sagittal plane. Following the tones from a metronome, subjects repeatedly raised and lowered their hands at a frequency of 1 Hz (Fig. 1B). All participants practiced the tasks for 3 to 5 min before the experiment.

Three different loads were applied for the lifting movements: 0 pounds (bare hands), 3 pounds (1.36 kg), and 15 pounds (6.80 kg). We chose 3 pounds because it was the entry-level weight for strength training [24], as well as 15 pounds both according to previous studies [25] and because it was the heaviest dumbbell that allowed subjects to complete the protocol without complaining of fatigue.

Movements were grouped in blocks. Each subject went through 12 blocks, and each block contained 12 to 14 movements (about 25 s) against a specific load magnitude. The subject was requested to rest for 40 s between blocks. The entire sequence of all blocks was randomized and lasted about 13 min. Each block was carried out as follows:

1. The experimenter reminded the subject of the correct dumbbell for the upcoming block.

2. The subject picked up the assigned dumbbell (if any).

3. The computer played a starting sound.

4. The subject performed lifting movements following the metronome tempo.

5. The computer played an ending sound.

6. The subject put down the dumbbell and rested.

Two requirements were imposed to ensure the movement consistency during the experiment: (a) The lifting must start from the resting posture, finish close by the armpit, and the elbow must be restricted within the sagittal surface as much as possible, and (b) the movement speed was regulated by having the subject follow the 1-Hz metronome. If the subject failed to meet any of the requirements, then s/he would be verbally reminded to correct the movement in the upcoming trial. We video recorded the whole experiment and visually checked the movement consistency. Sample video recordings are provided in the Supplementary Materials.

### Brain hemodynamic data acquisition using fNIRS

A multichannel fNIRS device (model ETG-4100, HITACHI Inc., Japan) was used to capture brain hemodynamic signals at a 10-Hz sampling rate. The device calculated the corresponding hemoglobin and deoxyhemoglobin density following the modified Beer–Lambert Law [26]. We identified contralateral Brodmann Area 4 (BA4) and BA6 as regions of interest (ROIs) because of their roles in motor execution (BA4) and motor planning (BA6) [27]. Moreover, they were picked as ROIs in previous studies [28].

**Fig. 2.**
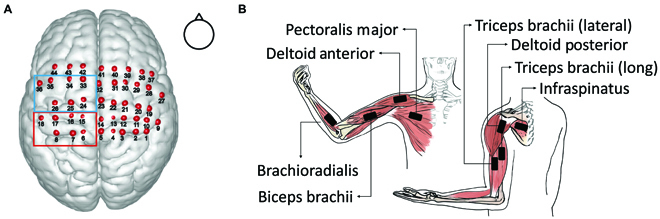
fNIRS and surface EMG configuration. (A) The spatial location of one representative subject. On the basis of the registration probability, channels in the red rectangle is located in BA4 (channels 6, 7, 8, 15, 16, 17, and 18), and channels in the blue rectangle is located in BA6 (channels 24, 25, 26, 33, 34, 35, and 36). (B) The placement of EMG sensors. Sensors were placed at the belly of the biceps, brachioradialis, pectoralis major, deltoid anterior, triceps lateral, deltoid posterior, triceps long, and infraspinatus under the supervision of an experienced therapist.

Laser optodes were configured using a high-density 3 × 10 probe with 16 emitters and 14 detectors to form 44 channels, which covered the BA4 and BA6 of both hemispheres (Fig. 1C). The probe was placed on the head following the international 10–20 system [29] to ensure coverage of the ROIs. Specifically, we first placed the probe over the subjects’ heads with channel 23 positioned at the CZ point. Next, we aligned the front brim of the probe with the coronal plane of the skull. A 3-dimensional magnetic space digitizer (Polhemus Patriot, Polhemus Inc., Vermont, USA) was used to measure the locations of the channels after the scan to determine the anatomical location of each channel. Figure 1D depicts the flow chart of the experiment within one visit. MNI (Montreal Neurological Institute and Hospital) coordinates of each channel were calculated using NFRI functions [30]. See Fig. 2A for probe configuration.

### Acquisition of surface electromyographic signals

Surface electromyographic signals were captured using the Trigno Lab Wireless EMG System (Delsys Inc., USA) at a 1,925.925-Hz sampling rate. The EMG sensors were placed on the bellies of 8 muscles: pectoralis major, anterior deltoid, posterior deltoid, biceps, triceps long head, brachioradialis, triceps lateral head, and infraspinatus. The placement of EMG sensors is shown in Fig. 2B.

### fNIRS signal processing

We used the NIRS-KIT toolbox (version 2.0) [31] to analyze the brain hemodynamic signals. Oxygen–hemoglobin (oxy-Hb) signals were used to quantify cortical activity in this study because they were sensitive to regional cerebral blood flow fluctuations [32]. The raw oxy-Hb signals were first detrended with the linear detrending method and then motion corrected using Temporal Derivative Distribution Repair; then, the signals were band-pass filtered with cutoff frequencies 0.01 and 0.08 Hz to remove physiological artifacts and high-frequency noise.

### Surface electromyographic signal processing

The raw surface electromyographic signals were band-pass filtered with the cutoff frequencies of 20 and 400 Hz, rectified, and low-passed at a cutoff frequency of 20 Hz to obtain the linear envelope [33]. All processing were performed offline by custom-written programs (MATLAB R2020b, MathWorks Inc.).

In this study, we extracted time-invariant muscle synergies using a non-negative matrix factorization algorithm [34]; synergies were calculated separately for each load condition. The synergy decomposition was conducted using the following equation:

$$M_{t,8}=T_{t,n}\cdot V_{n,8}+\text{residuals}$$ (1)

where *M* was the filtered EMG matrix with 8 columns of data; *t* was the time length of each block; *n* was the number of muscle synergy; *T* was the matrix of time profiles, in which each column contained the activation profiles corresponding to each row of vector in all trials; and *V* was the matrix of *n* synergy vectors, in which each row contained a combination of the 8 muscles with different weights and each vector was normalized to have unit length during factorization.

To evaluate the goodness of EMG reconstruction, the criterion of variance account for (VAF) [35] was adopted here in the following equation:

$$\mathrm{VAF}=100\times \left(1-\frac{{\left(\left\|M-D\right\|\right)}^{2}}{{\left(\left\|M-\text{mean}\left(M\right)\right\|\right)}^{2}}\right)$$ (2)

where *D* was the reconstructed EMG matrix and mean(*M*) represented the averaged EMG across 8 muscles at the same data point. We adopted the 95% criterion [36]; the number of synergy vectors (*k*) that sufficiently recaptured the original EMGs was then defined as the number (*n*) when VAF exceeded 95%. Thus, we extracted the 3-component synergy for each condition. The global VAFs were 98.47%, 97.50%, and 97.25% for 0, 3, and 15 pounds, respectively.

### Synergy similarity between load conditions

To quantify the overall similarity between the synergies of each load condition, cosine similarity was used to evaluate the degree of matching. A random subject (subject 03; female, 27 years old) was chosen as the baseline subject. Her muscle synergies under the 0-pound condition were used throughout the analyses as the basis for synergy similarity. The closeness between synergy vectors in each subject with the corresponding basis synergy used the following equation:

$$X_{V}\left(i.j\right)=\frac{V\left(j\right)\cdot V_{B}\left(i\right)}{\left|V\left(j\right)\right|\cdot \left|V_{B}\left(i\right)\right|}\;\left(i.j=1,2,\ldots ,k\right)$$ (3)

where *X_V_* was a closeness matrix of synergy vectors defined by cosine similarity of *V* and *V_B_*; *V* and *V_B_* were synergy vectors from an individual subject and baseline, respectively; and *k* was the number of synergies that was determined by VAF; in this study, *k* was set to 3. Then, the vector from the individual subject was paired to one from basis synergy following the maximal scalar product criterion [37]. The overall similarity *S_V_* was calculated on the basis of closeness. The detailed equations were provided in the Supplementary Materials. The methodology has been reported in a previous study [38].

### Statistical analysis

Linear mixed-effects models with random intercepts were calculated to examine the relationship between the muscle synergy similarities and load:

$$\text{similarity}=a_{0}+a_{1}\;\text{load}+e$$ (4)

where similarity is the dependent variable, load is the independent variable with the fixed coefficient of *a*_1_, the intercept *a*_0_ is marked as a subject-specific term in R to model the random effect due to repeated measures, and *e* is the error. Both similarity and load are continuous variables. Because similarity ranges between 0 and 1, we have applied Fisher’s *z* transformation [39] to meet the assumptions of normality (Shapiro test, *P* > 0.05) and homoscedasticity (Bartlett test, *P* > 0.05) on the dependent variable [40].

The general linear model procedures were used to characterize the hemodynamic response of oxy-Hb in all 44 channels under 3 conditions. Beta coefficients, of which the sign and magnitude indicate the direction (positive/negative) and intensity of change in oxy-Hb, were generated for each subject, channel, and condition. Beta coefficients are referred to interchangeably with cortical activation in this manuscript unless otherwise specified. The relationship between synergy similarity and cortical activation was analyzed using linear mixed-effects models as follows:

$$\text{similarity}=a_{0}+a_{1}\;\text{beta}+e$$ (5)

where similarity is the dependent variable, beta is the independent variable (muscle activities are normally regulated by the brain) with the fixed coefficient of *a*_1_, the intercept *a*_0_ is marked as a subject-specific term in R to model the random effect due to repeated measures, and *e* is the error. The significance level in the analyses was set at 0.05. All statistical analyses were performed using R (version 4.2.1). The models were fitted using the ‘lmer’ package [41], and the significances were calculated using the ‘lmerTest’ package [42]. The conditional *r*^2^ was calculated using the ‘MuMIn’ package [43]; conditional *r*^2^ is the proportion of total variance explained through both fixed and random effects in the general linear model.

## Results

### Effects of load inertia on muscle synergies

**Fig. 3.**
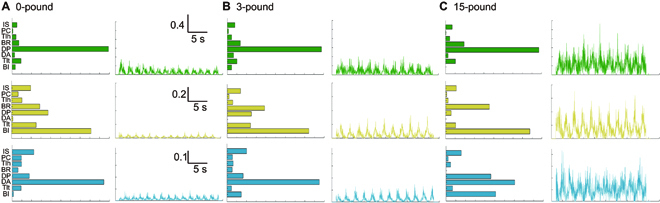
Muscle synergy extracted from an individual subject. (A) The synergy vectors and the corresponding time profiles under 0-pound condition. (B) The synergy vectors and the corresponding time profiles under 3-pound condition. (C) The synergy vectors and the corresponding time profiles under 15-pound condition. Muscles from top to bottom: IS, infraspinatus; PC, pectoralis major; Tlh, triceps long head; BR, brachioradialis; DP, deltoid posterior; DA, deltoid anterior; Tlt, triceps long head; BI, bicep. The different colors represent the different muscle synergies.

Non-negative matrix factorization decomposed raw EMGs into time-invariant synergy vectors and time-variant time profiles, known as muscle synergy. The representative muscle synergies are plotted in Fig. 3. As can be seen, in the first synergy (green), posterior deltoid shows the highest contribution under all 3 conditions; in the second synergy (yellow), the contributions are more evenly distributed with the largest influence from biceps brachii; and in the third synergy (blue), the anterior deltoid shows the highest contribution. With higher load inertia, it can also be seen that the activation levels of muscle synergies increase with load.

**Fig. 4.**
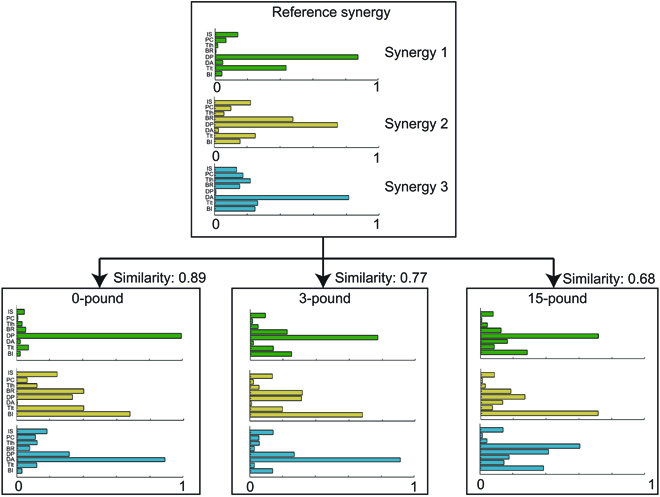
Similarity calculation. Muscle synergy extracted from 0 pounds of subject 03 was selected as the basis synergy. All muscle synergies were sorted by VAF in descending order. The overall similarity equals the sum of closeness, weighted by the contribution of the eigenvalue of the corresponding reconstructed EMG matrix.

Synergy vector similarities were calculated between each extracted muscle synergy and the basis synergy; the representative calculation of similarity is shown in Fig. 4. Higher similarity means that the synergy vectors are generally closer to the basis.

**Fig. 5.**
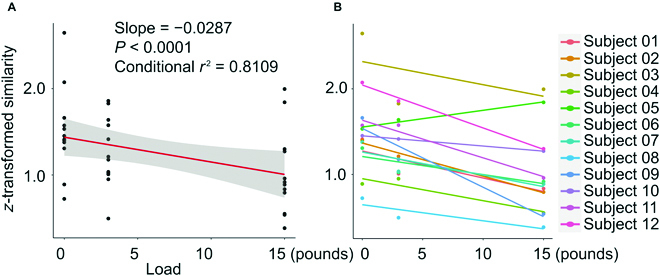
The effect of loads on the synergy similarities was fitted using a linear mixed-effects model with random intercepts. The corresponding P value and the conditional *r*^2^ from the linear model were plotted above the regression line. Significantly negative correlations were identified, indicating that motor control strategies vary among different load magnitudes. (A) The sample regression line of all subjects displays the overall tendency. (B) The sample regression lines of each subject to display the pooled data.

The relationship between load magnitude and synergy similarity was analyzed using a linear mixed-effects model. As shown in Fig. 5, a significant linear correlation was discovered with negative slopes, meaning that the *z*-transformed similarity decreased by 0.0287 for each pound (conditional *r*^2^ = 0.81, *P* < 0.0001).

### Relationship between muscle synergies and cortical activation (channel-wise)

**Fig. 6.**
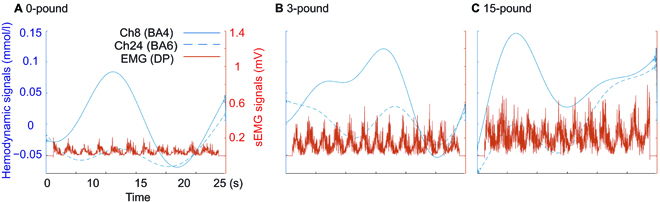
Aligned surface EMG and fNIRS hemodynamic signals from one representative subject. The hemodynamic signals of channel 8 (located in BA4), channel 24 (located in BA6), and the linear envelopes of the posterior deltoid (the main agonist) under 0-pound (A), 3-pound (B), and 15-pound (C) conditions are plotted in the dual *Y* axis chart; the left *Y* axis represents the oxygenated hemoglobin density and the right *Y* axis represents the surface EMG (sEMG) signal.

The EMG and fNIRS signals were aligned to capture changes in both peripheral and central nervous systems. The combined signals under 0, 3, and 15-pound conditions are shown in Fig. 6. Increasing load inertia induced higher amplitude of surface EMG and hemodynamic signals.

**Fig. 7.**
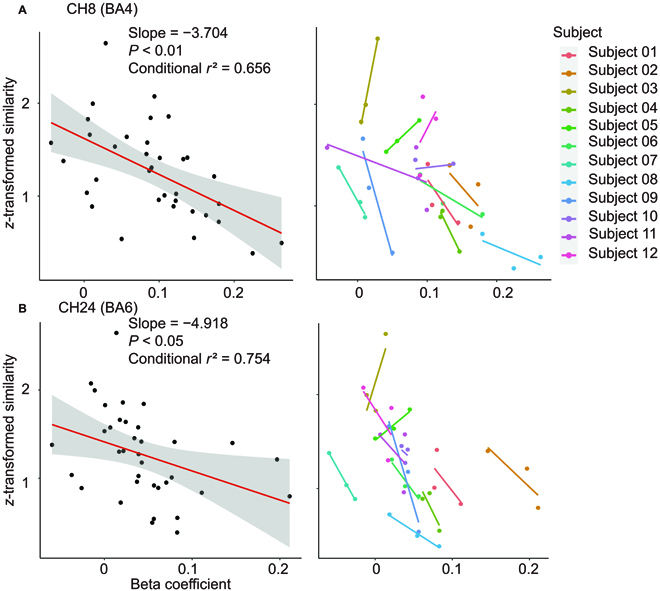
Correlation between synergy similarity and beta. The channels with the lowest *P* value in each ROI were selected as representative channels. We plotted the simple regression line to display the overall tendency. The linear relationships between synergy similarity and beta were fitted using a general linear model (random intercepts). Significantly negative correlations were identified, indicating that load magnitude affects both the central neural and muscular systems. (A) Channel 8, located at BA4. (B) Channel 24, located at BA6.

Linear mixed-effects models with random intercepts were used to analyze the relationship between *z*-transformed synergy similarity and beta coefficients of each channel. There were 4 of 7 channels (CH7, 8, 15, and 18) located at BA4 that show significantly linear correlations between *z*-transformed synergy similarity and beta coefficients with a negative slope (*P* < 0.05). There were 4 of 7 channels (CH24, 25, 26, and 36) located at BA6 that show significant linear correlations between synergy similarity and beta coefficients (*P* < 0.05). Representative channels are displayed in Fig. 7.

Table showed the details of the estimated slope, *t* value, *P* value, conditional *r*^2^, and location of significant correlations.

**Table.** Statistical details of significant correlations.

Channel	Estimated slope	*T* value	*P* value	Conditional *r*^2^	Location
CH7	−3.194	−2.58	0.016	0.722	BA4
CH8	−3.704	−3.07	0.005	0.656	BA4
CH15	−6.088	−2.30	0.029	0.691	BA4
CH18	−4.231	−2.38	0.024	0.662	BA4
CH24	−4.918	−2.73	0.011	0.754	BA6
CH25	−3.447	−2.59	0.020	0.757	BA6
CH26	−4.372	−2.28	0.036	0.644	BA6
CH36	−5.048	−5.05	0.012	0.650	BA6

### Relationship between muscle synergies and cortical activation (ROI-wise)

**Fig. 8.**
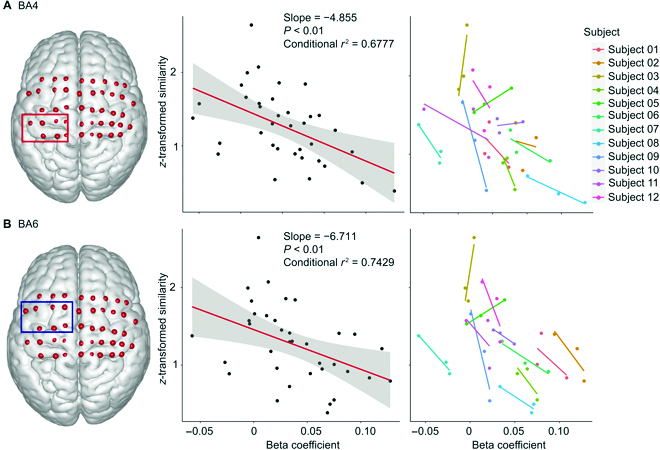
ROI-wise correlation between muscle synergy similarity and cortical activation. We plotted the simple regression line to display the overall tendency. The linear relationships between synergy similarity and beta were fitted using a general linear model (random intercepts). (A) The correlation in BA4. (B) The correlation in BA6.

Linear mixed-effects models with random intercepts were applied to analyze the relationship between synergy similarity and beta coefficients in both ROIs. As shown in Fig. 8A, there was a significant correlation between similarity and beta; the *z*-transformed synergy similarity decreased by 4.855 per unit of beta (conditional *r*^2^ = 0.6777, *P* < 0.01) in BA4. Regression lines of each subject were displayed to show individual trends.

In BA6, the *z*-transformed synergy similarities decreased by 6.711 per unit of beta (conditional *r*^2^ = 0.7429, *P* < 0.01), and BA6 showed a stronger linear trend than BA4, as suggested by the linear model (Fig. 8B).

## Discussion

In this study, we investigated load-induced changes in the peripheral and central nervous systems using fNIRS and surface EMG. Subjects were instructed to lift their right hands to the armpit against 3 different loads: 0 pounds (bare hand), 3 pounds, and 15 pounds. Significant changes in both peripheral and central nervous activities were detected: Similarities of synergy vector decrease with the increasing load, while cortical activations increase with load magnitude (the details are shown in Figs.S4 and S5). Moreover, a significant correlation between hemodynamic signals and synergy vector similarities was identified, indicating load-specific motor control strategies.

Our results are consistent with previous findings that muscle synergies are influenced and improved by biomechanical systems [19]. Specifically, various biomechanical properties, such as limb weight, joint torque weighting, and external constraints, may foster subject-specific synergy vectors [44,45]. On top of these studies, we have demonstrated that load magnitude alone is adequate to incur detectable changes in synergy vectors. Our data also replicated an earlier finding that the fNIRS activation in motor-related cortices correlates positively to load magnitude [22], supporting the idea that the human motor cortex plays a crucial role in the formation of synergy vector [46].

A linear correlation has been identified between cortical response and changes in synergy vectors, which is consistent with the previously reported central–peripheral coherence for small-extent movements. Specifically, both EMG and brain activity were up-regulated by the increased load during hand grip [47], finger pushing [48], and isometric elbow contraction [49]. In this study, we contribute to the literature that the central–peripheral correlation also exists in large-extent movements; moreover, our data suggest that a common modality of training, dumbbell weight, may simultaneously modulate the central and peripheral activity. This feature would be convenient for the justification of weight dosage during training.

Many studies seem to agree that synergy vectors remain robust in movements with similar kinematics, meaning that differences in EMG across tasks are explained by various time profiles but identical synergy vectors [50,51]. However, our study proposes a different possibility that synergy vectors may indeed change, even when kinematic requirements are identical across conditions. We have demonstrated that the relative change in synergy vector is correlated with load inertia, illustrating the possibility that synergy vectors may vary across tasks. The difference between our findings and the existing literature may be explained by 2 factors. First, the metrics used in previous studies to compare synergy vectors (e.g., number of synergies, the VAF, number of shared synergies, etc.) may not be detailed enough to describe subtle differences in the vectors. Second, it is possible that mechanical loading evokes a different strategy of movement control [52]. This latter possibility is particularly interesting because our data suggest that the adoption of a new strategy is likely a gradual process, rather than an abrupt shift.

In terms of clinical significance, this study potentially helps clinicians understand how the nervous and muscular system reacts to progressively heavier load, which is commonly used in clinical rehabilitation [53]. The study protocol has the possibility to be expanded to a clinical procedure that combines traditional weight training, together with quantitative metrics on the progress of recovery. This possible usage, nonetheless, needs substantial testing in the patient population before actual practice. In patient experiments, individualized adaptation of the protocol may be necessary, as not all patients may be capable of lifting their arms while holding dumbbells of varying weights.

Our results showed that adding inertial load during large-extent upper limb movements could progressively alter the muscle vector. Moreover, the synergy similarity to a common basis decreased with higher activation in BA4 and BA6. The effects of added inertia, including the decrease of synergy similarity and increase of motor cortical activity, potentially provide a new benchmark for therapeutic goal setting.

## Data Availability

The datasets used and/or analyzed during the current study are available from the corresponding authors on reasonable request.

## SUPPLEMENTARY MATERIALS

Supplementary 1 Similarity calculation Activation analysis of hemodynamic signals Figs. S1 to S5 Tables S1
