# Supplementary material for: Heavier Load Alters Upper Limb Muscle Synergy with Correlated fNIRS Responses in BA4 and BA6
Source: Cyborg Bionic Syst. 2023 May 31;4:0033. doi: 10.34133/cbsystems.0033 (PMC10233656; doi:10.34133/cbsystems.0033)
Supplement: Supplementary 1 — Similarity calculation Activation analysis of hemodynamic signals Figs. S1 to S5 Tables S1 [file cbsystems.0033.f1.zip › Supplementary Materials.docx]

Supplementary Materials

Similarity calculation

To quantify the overall similarity between the synergies of each load condition, cosine similarity was used to evaluate the degree of matching as in our previous study (Niu et al. 2022). A random subject (subject03) was chosen as the reference subject. Her muscle synergies in the 0-pound condition were used throughout the analyses as the basis for synergy similarity. The similarity between synergy vectors in each subject with the corresponding reference synergy used the following equation:

$$\begin{aligned} X_{V}\left( i.j \right)=\frac{V\left( j \right) \cdot V_{B}\left( i \right)}{\left| V\left( j \right) \right|\cdot\left| V_{B}\left( i \right) \right|} \left( i.j=1,2,\ldots,k \right) \#\left( 1 \right) \end{aligned}$$

Where $X_{V}$ was a closeness matrix of synergy vectors defined by cosine similarity of $V$ and $V_{B}$, $V$ and $V_{B}$ were synergy vectors from individual subject and reference respectively, $k$ was the number of synergies which determined by VAF, in this study, $k$ was set to 3.

Therefore, each column of $X_{V}$ consisted of closeness between a vector from an individual subject and the $k$ vectors in reference synergy, and the vector from the individual subject was then paired to one from baseline synergy when they had the maximal closeness among the $k$ values following the maximal scalar product criterion (Cheung 2005).

$$\begin{aligned} N\left( i \right)=Max\left( X_{V}\left( 1,i \right),X_{V}\left( 2,i \right),\ldots,X_{V}\left( k,i \right) \right)\left( i=1,2,\ldots,k \right)\#\left( 2 \right) \end{aligned}$$

$$\begin{aligned} C_{V}\left( i \right)=X_{V}\left( N\left( i \right),i \right) \left( i=1,2,\ldots,k \right) \#\left( 3 \right) \end{aligned}$$

where operator “$Max$” was meant to obtain the maximal value of closeness among the $i^{th}$ column of $X_{V}$. $C_{V}$ was then defined as closeness of individual vector.

$$\begin{aligned} \left[ e_{1}{,e}_{2},\ldots,e_{k} \right]=pca\left( C \right) \#\left( 4 \right) \end{aligned}$$

$$\begin{aligned} \lambda_{i} = \frac{e_{i}}{\sum_{j=1}^{k} e_{j}} \left( i=1,2,\ldots,k \right) \#\left( 5 \right) \end{aligned}$$

$$\begin{aligned} S_{V}=\sum_{i=1}^{k} \lambda_{i} \cdot C_{V}\left( i \right) \left( i=1,2,\ldots,k \right)\#\left( 6 \right) \end{aligned}$$

Where $C$ was the time profiles, $pca$ (Principal component analysis) calculated the eigenvalue ($e$) of each synergy in $C$. $S_{V}$ was the overall similarity weighted by contribution of each eigenvalue. Since $similarity$ ranged between 0 and 1, we applied Fisher’s z transformation (Silver and Dunlap 1987) to meet the assumptions of normality (Shapiro test, p > 0.05) and homoscedasticity (Bartlett test, p > 0.05) on the dependent variable.

Activation analysis of hemodynamic signals

The overview of the activation in the ROIs map, interpolated from the averaged beta coefficients, is shown in Fig.S1. As in our previous study, we performed the activation analysis in both channel-wise and ROI-wise. If the beta coefficient were significantly greater than 0 in one-sample two-tailed t-tests and survived the FDR (false discover rate) correlation (multiple correlations only applied in channel-wise activation), the corresponding channel or ROI was deemed activated.


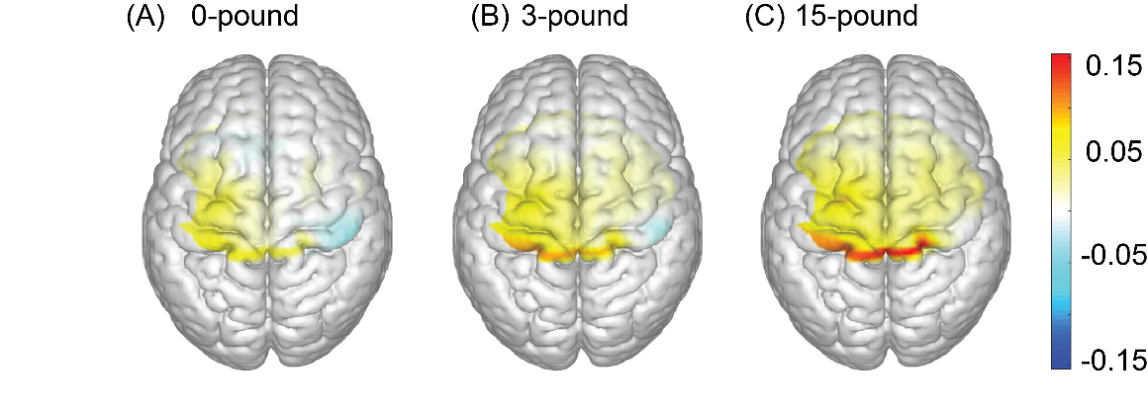


S1. Averaged beta on a standardized brain model (n = 12). (A) The 0-pound condition, in which a typical contralateral activation pattern was observed. (B) The 3-pound condition, in which BA4 and BA6 show higher activation levels. (C) The 15-pound condition, in which BA4 and BA6 exhibited the highest activation among the three conditions.

In the 0-pound condition (Fig. S2A), 3 of 7 channels (7,8,18, all adjusted P < 0.05) in BA4 were activated, 1 of 7 channels (26, adjusted P < 0.05) in BA6 were activated. In the 3-pound condition (Fig.S2B), all channels in BA4 were activated (all adjusted P < 0.05), and 5 of 7 channels (24,25,26,34,36, all adjusted P < 0.05) were activated. In the 15-pound condition (Fig.S2C), all channels in BA4 were activated (all adjusted P < 0.05), and 6 of 7 channels in BA6 were activated (24,25,26,34,35,36, all adjusted P < 0.05).


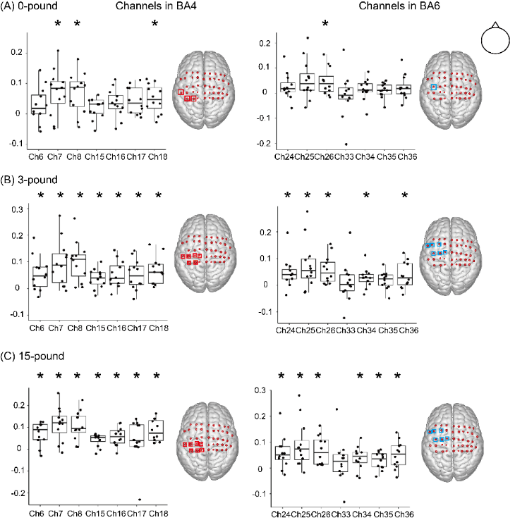


S2. Channel-wise activation. Box-plot shows the median and percentile beta value of channels located in ROIs, and jitters show the beta value of each subject. (A) The 0-pound condition. 3 out of 7 channels were activated in BA4 and 1 out of 7 channels was activated in BA6. (B) The 3-pound condition. All channels in BA4 were activated, and 5 of 7 channels were activated in BA6. (C) The 15-pound condition. All channels in BA4 were activated, and 6 of 7 channels were activated in BA6. * FDR-adjusted p < 0.05 from one-sample t-test.

To improve the spatial consistency across channels, we employed a group analysis based on ROI (Okamoto et al. 2009). The 3 channels with the highest probability of registration likelihood were chosen for the weight adjustment of signals based on ROI. Therefore, the weight-adjusted hemodynamic signal for each ROI was:

$$\begin{aligned} oxyHb_{ROI}=\frac{\sum_{i=1}^{3} P_{i}*oxy_{i}}{\sum_{i=1}^{3} P_{i}} \#\left( 7 \right) \end{aligned}$$

Where $oxyHb_{ROI}$ represented the oxy-Hb signals of each ROI, and $P_{i}$ represented the likelihood of channel$i$out of 3, ranked by the likelihood in the probabilistic registration.

As shown in Fig.S3.

In the 0-pound condition, BA4 was significantly activated (BA4: t11 = 2.2323, p < 0.05; BA6: t11 = 1.9417, p = 0.07822). In the 3-pound condition, both BA4 and BA6 were significantly activated (BA4: t11 = 3.8592, p < 0.01; BA6: t11 = 3.4028, p < 0.01); In the 15-pound condition, the activation was also significant in both BA4 and BA6 (BA4: t11 = 4.2181, p < 0.01; BA6: t11 = 3.8826, p < 0.01).


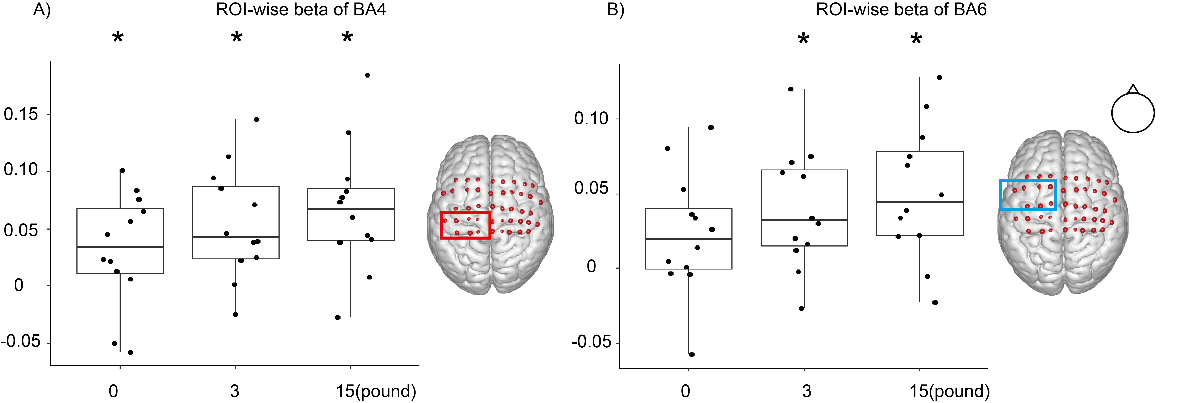


S3. ROI-wise activation. BA4 and BA6 were both activated in 3-pound and 15-pound conditions. ROI-based group analysis failed to detect cortical activation of BA6 in the 0-pound condition. * p < 0.05, indicating beta-values were significantly greater than 0.

Correlation analysis of hemodynamic signals

We performed both channel-wise and ROI-wise analysis to test the relationship between the beta coefficients and the magnitude of load inertia. 3 of 7 BA4-channels exhibit statistically significant positive correlations between load magnitudes and beta coefficients (p < 0.05); all BA6-channels exhibit significant positive correlations between loads and beta coefficients (p < 0.05). Fig.S4 shows the linear relationships from the most significant channels in both ROIs. The channel-wise details of slope, T-value, P-value, and location are listed in Table.S1.


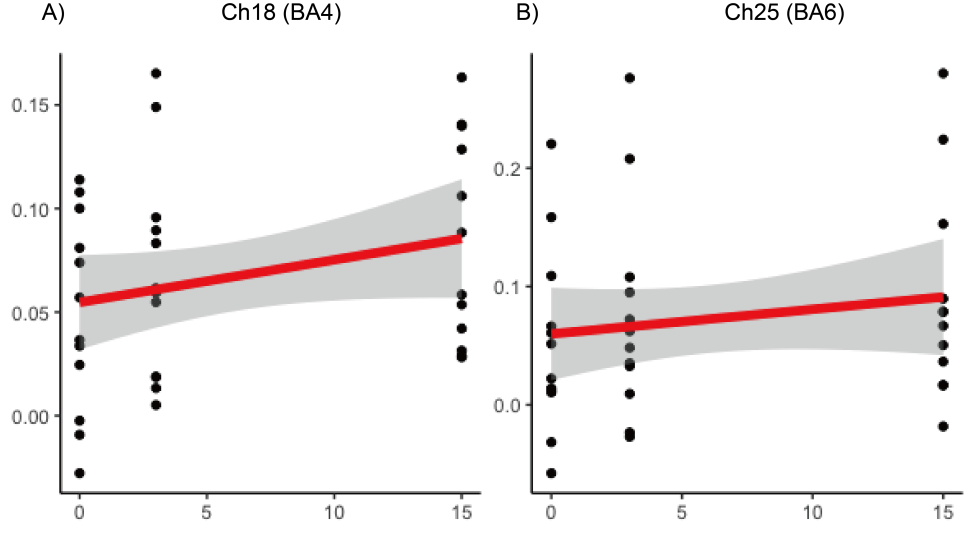


S4. Representative examples of channel-wise correlation. Channel 18 was located in BA4, and channel 25 was located in BA6. Representative channels were chosen based on the p-values of the mixed-effects model. We plotted the sample-regression line to display the overall tendency.

Table S1. Statistical details of significant channels.

| Channel | Estimated slope | T-value | P-value | Conditional r-square | location |
| --- | --- | --- | --- | --- | --- |
| Ch6 | 0.00251 | 3.680 | 0.001 | 0.809 | BA4 |
| Ch7 | 0.00252 | 4.020 | 0.001 | 0.910 | BA4 |
| Ch18 | 0.00205 | 4.299 | 0.000 | 0.872 | BA4 |
| Ch24 | 0.00176 | 4.443 | 0.000 | 0.930 | BA6 |
| Ch25 | 0.00208 | 5.401 | 0.000 | 0.970 | BA6 |
| Ch26 | 0.00124 | 3.805 | 0.001 | 0.946 | BA6 |
| Ch33 | 0.00194 | 3.731 | 0.001 | 0.941 | BA6 |
| Ch34 | 0.00134 | 4.275 | 0.000 | 0.917 | BA6 |
| Ch35 | 0.00110 | 3.662 | 0.001 | 0.877 | BA6 |
| Ch36 | 0.00162 | 3.944 | 0.001 | 0.902 | BA6 |

We run a linear model in each ROI to examine the effect of load magnitudes on cortical activation. As shown in Fig.S5A, we found significantly positive correlations between load magnitudes and cortical responses. The beta coefficient increased by 0.001994/pounds (conditional r2 = 0.8534, p<0.001) in BA4. In Fig.S5B, the activation level of BA6 also significantly correlated with the magnitude of load by 0.001564/pounds (conditional r2 = 0.9335, p<0.0001), BA6 show a stronger linear trend than BA4, as suggested by the mixed-effect linear model.


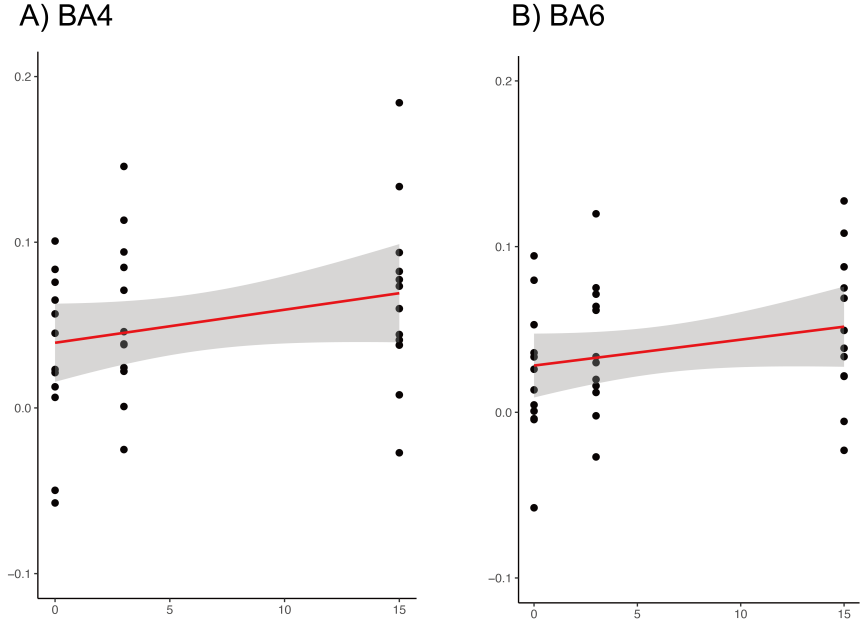


S5. ROI-wise correlation between beta coefficients and weight. We plotted the sample-regression line to display the overall tendency in both BA4 (A) and BA6 (B). Effects of load on the cortical activation level were fitted using mixed-effects linear models. Significantly positive correlations were found in both ROIs.

Reference:

Cheung, V. C. K. 2005. ‘Central and Sensory Contributions to the Activation and Organization of Muscle Synergies during Natural Motor Behaviors’. *Journal of Neuroscience* 25 (27): 6419–34. https://doi.org/10.1523/JNEUROSCI.4904-04.2005.

Niu, Chuanxin M., Chih-Hong Chou, Yong Bao, Tong Wang, Lin Gu, Xiao Zhang, Lijun Cui, et al. 2022. ‘A Pilot Study of Synergy-Based FES for Upper-Extremity Poststroke Rehabilitation’. *Neuroscience Letters*, April, 136621. https://doi.org/10.1016/j.neulet.2022.136621.

Okamoto, Masako, Daisuke Tsuzuki, Lester Clowney, Haruka Dan, Archana K. Singh, and Ippeita Dan. 2009. ‘Structural Atlas-Based Spatial Registration for Functional near-Infrared Spectroscopy Enabling Inter-Study Data Integration’. *Clinical Neurophysiology* 120 (7): 1320–28. https://doi.org/10.1016/j.clinph.2009.01.023.

Silver, N. Clayton, and William P. Dunlap. 1987. ‘Averaging Correlation Coefficients: Should Fisher’s z Transformation Be Used?’ *Journal of Applied Psychology* 72 (1): 146–48. https://doi.org/10.1037/0021-9010.72.1.146.
